# Changing trends in the epidemiology and surgical treatment of benign parotid gland tumours: a 10-year retrospective comparison in a tertiary referral centre of southeast Bavaria

**DOI:** 10.1186/s13005-026-00618-w

**Published:** 2026-04-15

**Authors:** Ioannis Michaelides, Luisa Symeou, Joshua Wällisch, Johannes Meier, Christopher Bohr, Julian Künzel

**Affiliations:** 1 Department of Otorhinolaryngology, Head and Neck Surgery, University Hospital of Regensburg, Franz-Josef-Strauss-Allee 11, Regensburg, 93053 Germany; 2 Department of Oral and Maxillofacial surgery, University Hospital of Regensburg, Franz-Josef-Strauss-Allee 11, Regensburg, 93053 Germany

**Keywords:** Benign salivary gland tumors, Warthin’s tumor, Pleomorphic adenoma, Cystadenolymphoma

## Abstract

**Background:**

Benign salivary gland tumors are in general rare. Pleomorphic adenomas (PA) and Warthin’s tumors (WT) make up to 90% of these tumors. Their epidemiology shows significant regional differences but also changes over time. In addition to that, new developments in the surgical approach, like extracapsular dissection (ED), have been introduced.

**Methods:**

We retrospectively analyzed epidemiological and clinical data of 347 patients (184 male and 163 female) treated in the ENT-Department of the University Hospital of Regensburg in 2010/11 and 2020/21 and assessed their changes over time.

**Results:**

WT is by far the most common benign tumor of the parotid gland, followed by PA and the age of diagnosis shows an increasing tendency. The hospitalization time showed a significant reduction after a period of ten years. Patients treated with ED are being hospitalized for a shorter period of time. Regular smokers tend to present more WT and are being diagnosed at younger age. Patients with WT have a higher body mass index (BMI) than other patients.

**Conclusions:**

Over the last decade, WT remained the most common benign parotid tumor in Southeast Bavaria, while the implementation of ED has potentially impacted clinical resource utilization coincide with reduced duration of inpatient hospitalization.

**Supplementary Information:**

The online version contains supplementary material available at 10.1186/s13005-026-00618-w.

## Introduction

Salivary gland tumors represent a highly heterogenous group of neoplasms comprising 39 distinct entities in the current WHO classification [1], of which 15 are classified as benign [2]. The parotid gland is the most frequently affected site by salivary gland tumors and pleomorphic adenoma (PA) and cystadenolymphoma (Warthin’s tumor, WT) are making up more than 80% of all tumors [3, 4]. A large German study including 1818 patients, reported that the most common benign neoplasm of the parotid gland is PA followed by WT, however a trend in favor of WT was observed [5]. On the other hand, another German study conducted by Psychogios et al., which also specifically examined benign tumors of the parotid gland, showed in contrary, that WT is the most common entity with 42% followed by PA with 29% [6]. The epidemiological data suggests significant regional differences, thus more locoregional studies are reasonable.

The slow but steady growth rate and the rare risk of malignant transformation of PA over time [7], make surgical removal the treatment of choice. An early treatment should be preferred as this approach often allows for limited surgery like an extracapsular dissection (ED), which leads to a favorable surgical outcome with reduced complications including less injuries of the facial nerve [8, 9]. The exact pathogenesis of both tumors is not yet clarified, but there is a high association between smoking and WT [7, 10]. On the other hand, sex is a relevant risk factor for both tumors with higher prevalence of PA among females and WT among males [11, 12]. The present study was conducted to shed light on the regional epidemiology of these tumors. Furthermore, we assessed the overtime changes of the incidence and hospitalization time as well as lifestyle factors of the most common entities. A regional update of parotid gland tumor epidemiology has a clinical impact, because patients with benign salivary gland lesions make up a relevant proportion of any Head and Neck department.

## Materials and methods

We retrospectively examined patients who received surgical treatment in the University Hospital of Regensburg ten years apart (2010/11 and 2020/2021) and were diagnosed with a benign lesion of the parotid gland. Clinical and demographic data regarding sex, smoking, weight and height (body mass index, BMI), surgical procedure like extracapsular dissection (ED), partial superficial parotidectomy (PP), lateral parotidectomy (LP) and total parotidectomy (TP), time of hospitalization, were collected according to the available medical documentation using SAP^®^ (© 2024 SAP SE) and documented in Microsoft Excel 16.82 (Microsoft Corporation, Redmond, WA, USA). The statistical analysis was performed with GraphPad Prism version 10.4.1 for MacOS (GraphPad Software, San Diego, California USA). For non-normally distributed data we used the Mann-Whitney U-test for two groups and Kruskal-Wallis test for more than two groups. We applied Fisher’s exact test for contingency tables, and Dunn’s test for multiple comparisons. P-values below 0.05 were considered statistically significant. We calculated the BMI of the patients according to the standard formula (BMI = weight (kg) / [height (m)] ^2^).

## Results

### Patient characteristics

347 patients (184 male and 163 female) with a mean age of (56.55 years ±13.92) treated in our clinic between 01.01.2010–31.12.2011 (cohort A) and 01.01.2020–31.12.2021 (cohort B) were included in this retrospective study. Because of the low numbers of other tumor entities, we focused on the two most common benign tumors of the parotid gland, namely WT and PA. 82 (23.34%) patients were treated with ED, 246 (71.18%) with PP and 19 (5.48%) received a TP (Fig. 2A).

### Epidemiology of benign salivary gland neoplasms over time

**Fig. 1:**
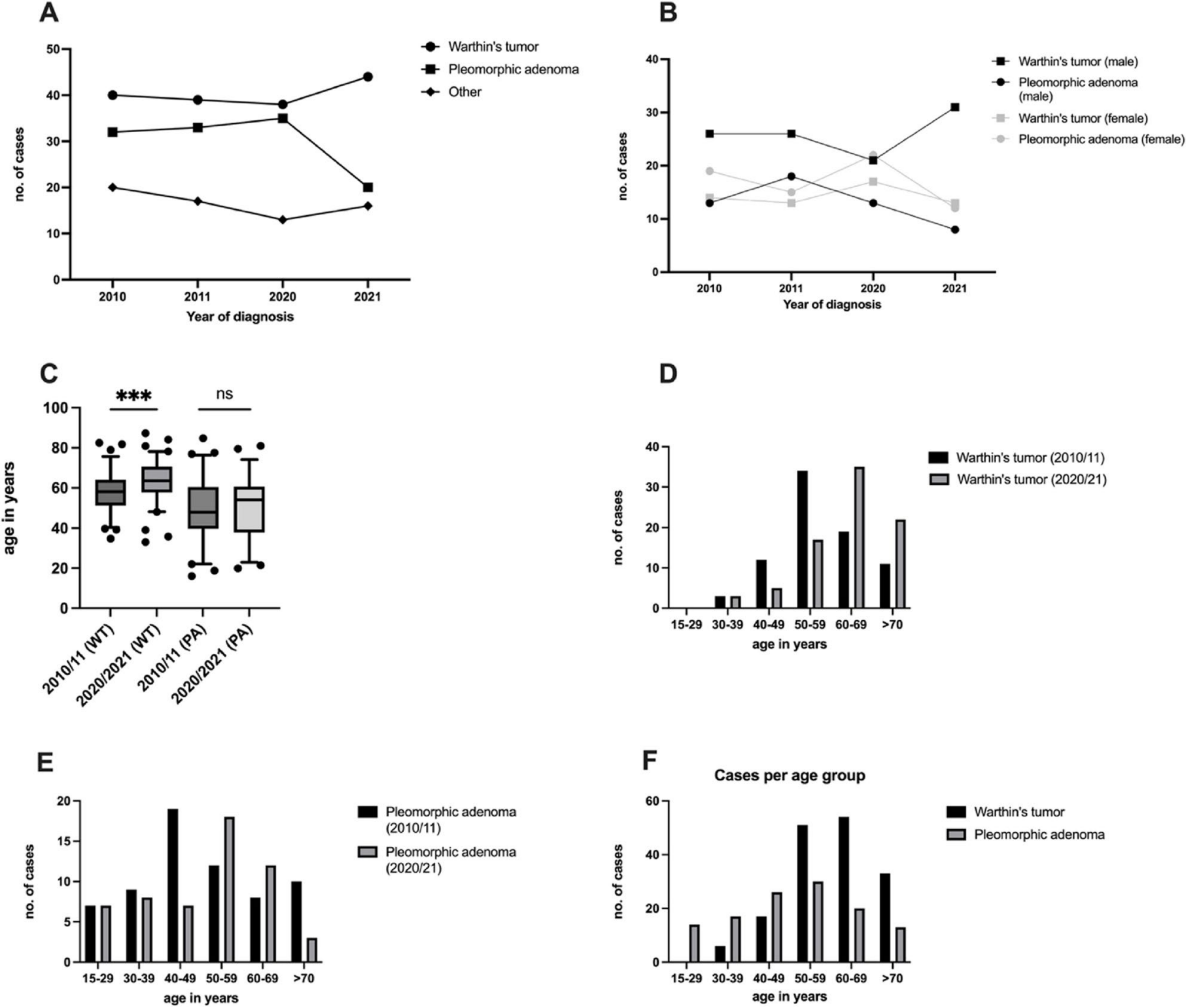
Number of cases per year (**A**), number of cases per year stratified by sex (**B**) age of diagnosis for WT and PA in each of the investigated years (**C**) appearance of WT (**D**) and PA (**E**) as well as comparison of both entities (**F**) in different decades of life and comparison between cohort A and B

Over the years 2010/2011 (cohort A) and 2020/21(cohort B) WT remained the most common diagnosed benign lesion of the parotid gland (46.4% overall), followed by PA (34.6% overall), (Table 1). In 2021 we observed a reduction in the newly diagnosed PA, but in general the count and proportions of the different entities remained stable after a decade. During the COVID-19 pandemic patients with tumors of the parotid gland were treated in our clinic as usual without any restrictions, which reflects in the stable number of new diagnoses when comparing cohorts A and B. Taking sex in consideration, we found that more male patients tend to develop WT (64.6%, *p* < 0.0001, two-sided, OR: 2.42), while they present less PA than females (56.67% vs. 43.33%, *p* < 0.012, two-sided, OR: 0.55) (Suppl. Tables 1 and 2). In addition to that, in year 2021, WT was also in the female population the most common diagnosed entity (Fig. 1B). Furthermore, we investigated the appearance of the different lesions in different age groups. Overtime we also observed an increase in the age of diagnosis in both entities. For WT patients the age increased from 57.89 (±10.32) to 63.5 (±10.22) years (*p* = 0.0003, two-tailed) and for PA patients from 49.58 (±16.33) to 50.31 (±14.63) years (*p* = 0.58, two-tailed) (Fig. 1C). We then examined in which decade of life each tumor tends to appear. Interestingly the appearance of both tumors was shifted up to the next decade of life in both cohorts (Fig. 1D and E). Lastly, we see that in younger people (ages below 50), pleomorphic adenoma is the most common benign tumor (Fig. 1F).

**Table 1:** List of the diagnosed benign salivary gland tumors and their incidence in our cohorts

Entity	Cohort A (2010-11), *n* (%)	Cohort B (2020-21), *n* (%)	Cohort A + B, *n* (%)
Warthin’s tumor	79 (43.65)	82 (49,4)	161 (46.4)
Pleomorphic adenoma	65 (35.91)	55 (33.13)	120 (34.58
Basal cell adenoma	12 (6.63)	4 (2,41)	16 (4.61)
Lymphoepithelial cyst	11 (6.08)	3 (1,81)	14 (3.75)
Lipoma	4 (2.21)	2 (1.2)	6 (1.73)
Lymphadenoma	2 (1.1)	0	2 (0.58)
Myoepithelioma	2 (1.1)	1 (0.6)	3 (0.86)
Onkcocytoma	2 (1.1)	4 (2.41)	6 (1.73)
Salivary duct cyst	2 (1.1)	2 (1.2)	4 (1.15)
Papillary cystadenoma	1 (0.55)	0	1 (0.29)
Pseudocyst	1 (0.55)	0	1 (0.29)
Epithelial cyst	0	8 (4.82)	8 (2.31)
Haemangioma	0	1 (0.6)	1 (0.29)
Oncocytic cyst	0	2 (1.2)	2 (0.58)
Retention cyst	0	1 (0.6)	1 (0.29)
Swannoma	0	2 (1.2)	2 (0.58)
Sum	181 (100)	166 (100)	347 (100)

### Hospitalization time

**Fig. 2:**
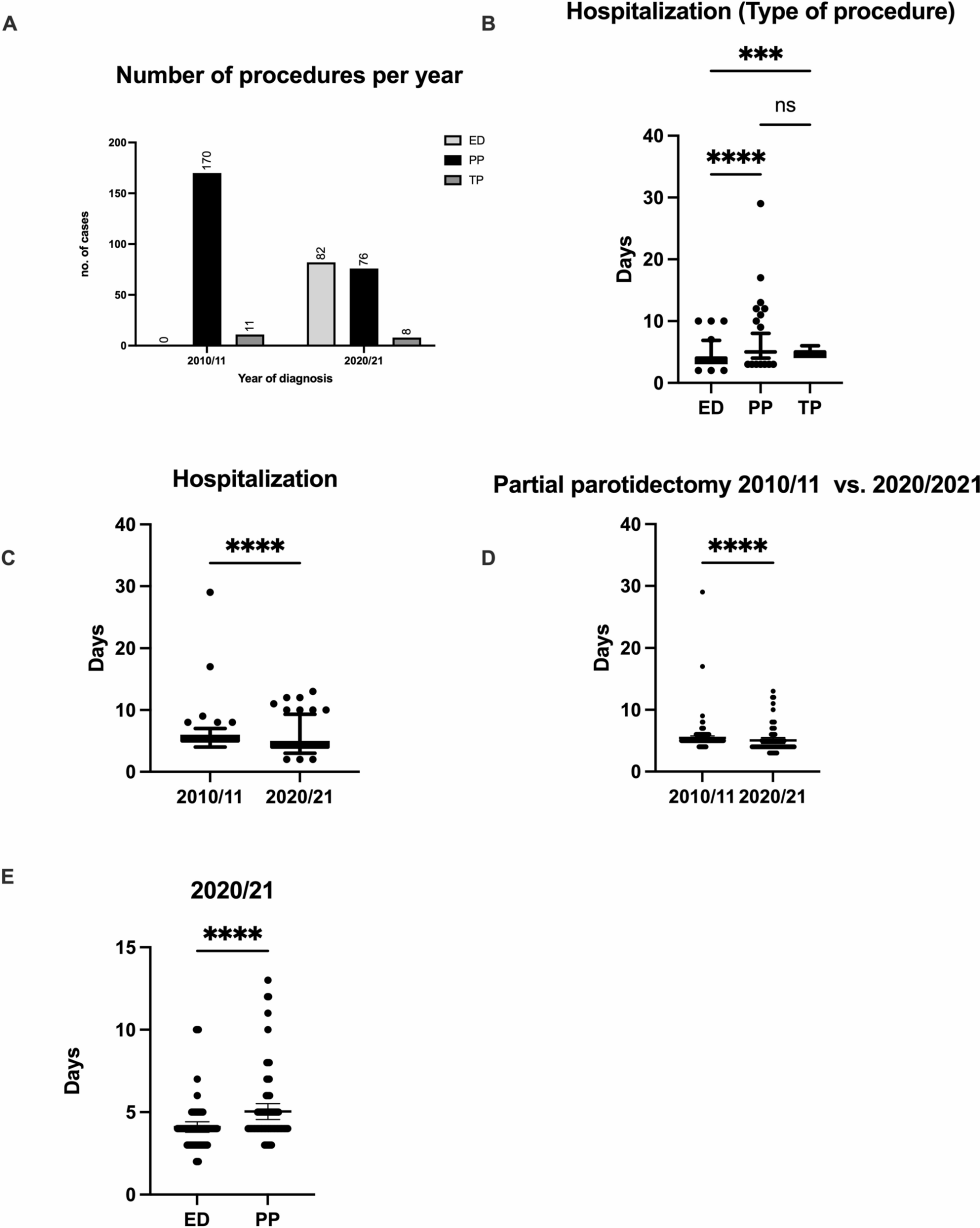
Number of each performed procedure within each cohort. **A** Comparison of mean hospitalisation days by procedure type (**B**) and cohort (**C**). Comparison of mean hospitalization after PP in both cohorts (**D**), comparison between ED and PP in cohort B (**E**)

The hospitalization time was investigated in both cohorts and stratified by the surgical procedure that was performed. We firstly compared the hospitalization time between the cohort A and B and found a significant reduction in the latter ( 5.41 vs. 4.55 days, *p* < 0.0001, two tailed) (Fig. 2A). Then the hospitalization times of all three procedures were analyzed and showed statistically relevant differences (*p* < 0.0001). The multiple comparisons revealed a highly significant reduction of the hospitalization time of patients on which an ED was performed, compared to PP or TP (ED vs. PP *p* < 0.0001, ED vs. TP *p* = 0.0004, Fig. 2B). Next, we compared the hospitalization times of PP in both cohorts. Patients treated in 2010/11 were hospitalized significantly longer than patients from 2020/21 (*p* < 0.0001, two-tailed) (Fig. 2C). In addition, the comparison of patients treated with ED or PP in cohort B revealed a significantly shorter hospitalization for patients with ED (*p* < 0.0001, two-tailed) (Fig. 2C).

### Lifestyle effects on appearance of WT and PA.

**Fig. 3:**
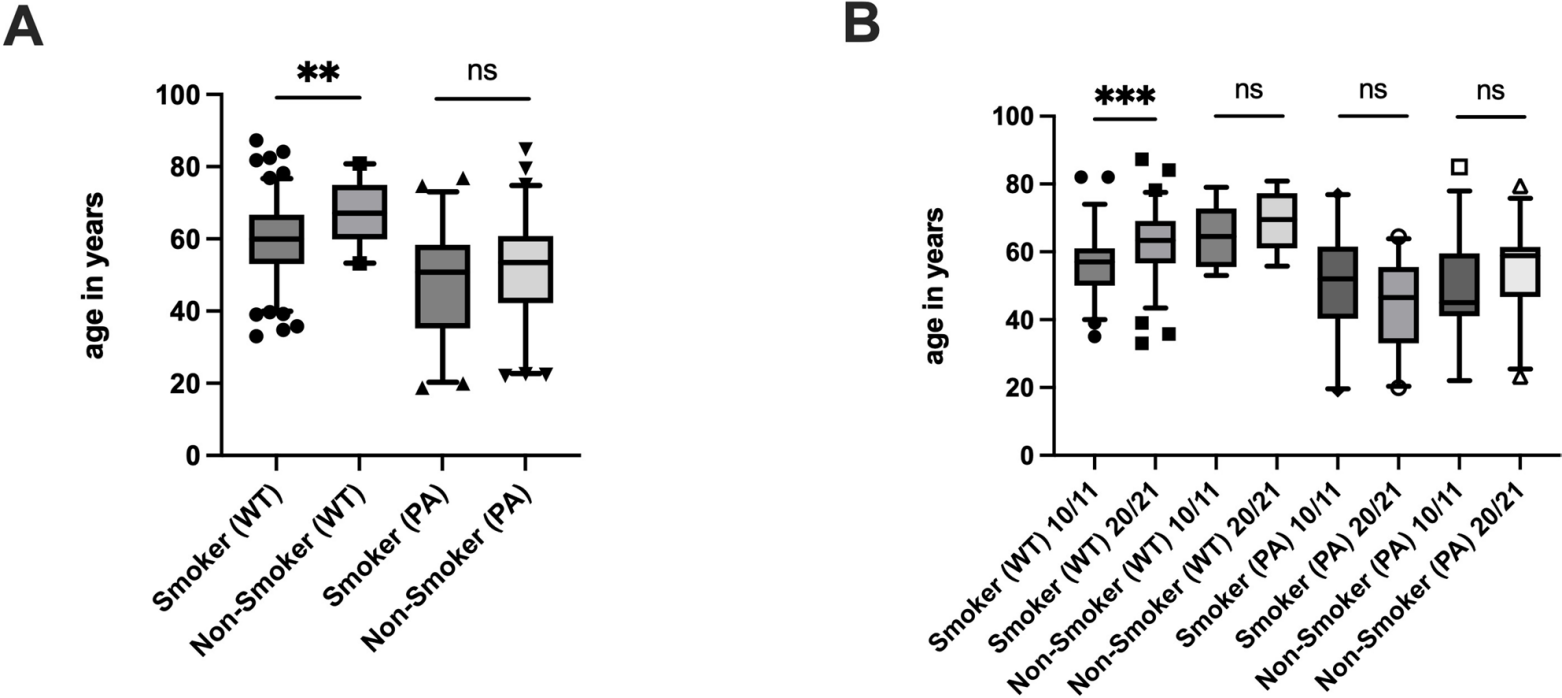
Comparison of age of diagnosis between smokers and non-smokers overall (**A**) and in the two cohorts (**B**)

Lifestyle factors like smoking, body-mass-index (BMI) were also evaluated. Overall, 60.5% of the patients were regular smokers, with an increasing tendency over time (52% vs. 62.9% in cohorts A and B) (Suppl. Table 3). More specifically, 67.4% of the WT patients and 22.6% of the PA patients smoked regularly. To investigate if tobacco had any effect on the appearance of WT and PA we compared the age of manifestation of the respective tumor. Regular smokers with WT were significantly younger (mean age: 59.8 years, ±10.6) than the non-smoking patients (mean age: 66.92 years ±8.9, two-tailed, *p* = 0.0053) (Fig. 3A). On the other hand, smoking patients with PA (mean age: 47.14 years, ±14.7) were also younger than non- smokers (mean age: 51.6 ± 14.7), but not significantly (two-tailed *p* = 0.1433; Fig. 3A). Over the years, we also observed a change in the age of diagnosis in patients with WT and regular tobacco use. Over this ten-year period the age of diagnosis has significantly increased (*p* = 0.0004) from a mean age of 56.53 years (± 9.95) in 2010/11 to 62.59 years (±10.4) in 2020/21 (Fig. 3B), even though the smoking habits did not significantly change over time (mean cigarette consumption 18.2/day±10 and mean pack-years 33.3±14.7 in 2010/11 vs. 16.72/day±8.1 and 37.8±15.2 packyears in 2021/2022) (Suppl. Tables 4 and 5). Next, we investigated if BMI is a relevant factor that affects the appearance of WT and PA. The fraction of WT patients with a BMI > 30 kg/m^2^ was clearly elevated (42.1%) in comparison to the PA patients (22.9%). We also compared the BMI of the patients of the two entities using the Kruskal-Wallis test, the difference was in favor of WT, significantly higher compared to patients with PA (*p* > 0.0001), but also compared to all other benign tumors (*p* = 0.0001) (Suppl Fig. 1). We then separated the patients into categories according to their BMI to assess if the BMI affects the age of appearance of WT and PA. This analysis did not reveal any statistically significant difference in the age of appearance of each of these tumors. However, we noticed a trend in patients with BMI below 25, which seem to get diagnosed with a PA at younger age (mean age: 47.0±16.0 years vs. 54.5±13.8 with BMI 25-29.9 vs. 51.0±14.9 with BMI > 30), but this difference was not statistically significant. (Suppl. Figure 2).

## Discussion

In the present study the epidemiologic changes of benign tumors of the parotid gland were assessed. As expected, PA and WT were the most common tumors with a cumulative incidence of around 80% consistent with the current literature [3, 5]. An even higher incidence was observed in a British study where PA and WT showed a cumulative incidence of over 90% with more than two times more PA than WT [13]. Since there are significant differences in the incidence of each tumor even within the same country as seen in the studies conducted by Psychogios et al. in 2020 and Luers et al. in 2016 [5, 6], we believe that this monocentric study, at a large tertiary referral center provides valuable insights into the evolving epidemiology of benign parotid neoplasms. The results showed that in southeast of Bavaria WT is by far the most common benign neoplasm of the parotid gland (46.4% WT vs. 34.6% PA). This observation was consistent in both time periods investigated (2010/11 vs. 2020/21).

According to the Bavarian statistic agency about 18% of the residents of Upper Palatinate, which is the main catchment area of our clinic, are smokers [14]. In the present study population, a high percentage of patients smoked regularly, namely around 60% overall, and this number was also increased by almost 10% in the period analyzed, primarily because of the abundance of WT patients. More specifically, over two thirds of patients with WT were smokers in contrast to 22% of PA patients. Additionally, smokers were diagnosed with WT at significantly younger age (59.8 vs. 66.9 years), which leads to the assumption that smoking has a relevant impact on the appearance of WT. The exact pathomechanism is not yet clarified. Smokers tend to be diagnosed with PA at a younger age (47.1 vs. 51.6 years) as well. However, the difference was not statistically significant, but indicated a trend, that smoking might be a factor that affects the appearance of PA, even though no clear evidence about a connection between PA and smoking could be found in the literature [15]. There is evidence that there might be a connection between salivary gland tumors and BMI. A study showed that the prevalence of obesity (BMI > 30) and Diabetes mellitus Type II was significantly higher in patients with salivary gland tumors [16]. In the present study cohorts a much higher percentage of WT patients were obese (42.1%) in comparison to the PA patients (22.9%). At the same time both groups showed a higher obesity rate than the average population in Upper palatinate (16%) [14]. These data suggest that a correlation between benign parotid gland tumors and obesity may exist especially for WT, this however needs further investigation. Furthermore, patients with PA and a BMI < 25 are diagnosed at an earlier age.

In times of limited resources, hospitalization time has a high health-economic impact [17, 18]. The analysis of changes in hospitalization time revealed a significant decrease in cohort B (5.41 vs. 4.55 days, *p* < 0.0001). Firstly, the increasing pressure on the health care system to reduce costs, e.g. due to timely discharge and process optimization and secondly, the introduction of ED (after 2011 in the study center) are the most probable reasons. The stratification according to the type of surgical procedure confirmed this assumption, as patients treated with ED were hospitalized for a significantly shorter period of time in comparison to patients treated with partial or total parotidectomy (4,09 vs. 5.03 days, *p* < 0.0001). Even though the difference seems to be small, a reduction of the hospitalization of approximately one day, can have a huge impact as it releases a significant amount of resources and reduces the cost of treatment. More than that, several studies demonstrate that ED does not lead to increased surgical complications, in particularly facial nerve injury, and may even reduce the risk of other complications [8, 9]. While ED is an effective approach for appropriately selected patients [19], the potential necessity for surgical escalation requires a high level of clinical expertise. Mastery of the entire spectrum of parotid surgical techniques is essential to ensure patient safety and optimal outcomes when intraoperative difficulties necessitate a deviation from the primary surgical plan of ED [20].

The retrospective character of the study poses as a limitation. Smoking behavior is not always reliably assessed in a retrospective setting and sometimes not well reported. As stated before, the economic pressure on hospitalization time is confounding the effect of the less invasive surgical technique of ED. As salivary gland tumors are rare in general monocentric studies are of limited power, but the size of this sample and power of results respectively, deliver a valid picture about the situation in the southeast Bavarian area.

## Conclusion

In southeast Germany, WT is by far the most common benign parotid tumor followed by PA. The implementation of extracapsular dissection has optimized the surgical management of benign parotid tumors, serving as a primary driver in reducing hospitalization time. Additionally, environmental and lifestyle factors, notably smoking and obesity, appear to influence earlier onset and overall incidence, highlighting the need for further pathophysiological research.

## Supplementary Information

Supplementary Material 1.

## Data Availability

The datasets presented in this article are not freely available because of patient confidentiality and participant privacy terms.
