# Supplementary figures and images for: Changing trends in the epidemiology and surgical treatment of benign parotid gland tumours: a 10-year retrospective comparison in a tertiary referral centre of southeast Bavaria
Source: Head Face Med. 2026 Apr 15;22:43. doi: 10.1186/s13005-026-00618-w (PMC13088733; doi:10.1186/s13005-026-00618-w)

Suppl. Tbl. 2

Suppl. Tbl. 1


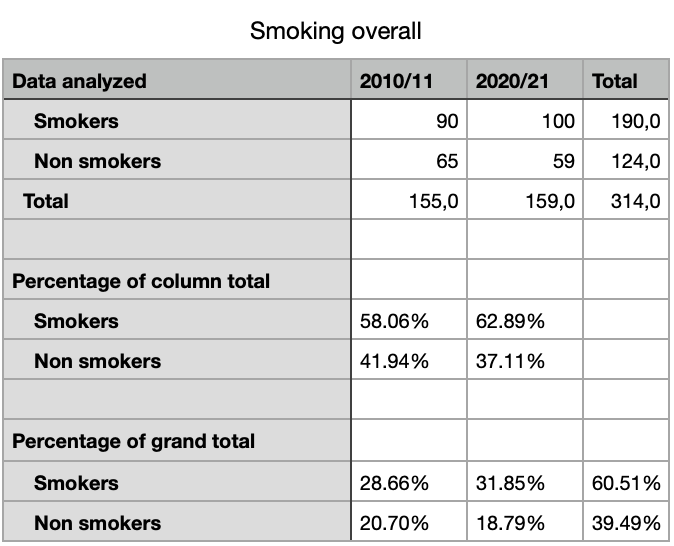


Suppl. Tbl. 3

Suppl. Tbl. 4

Suppl. Tbl. 5

Suppl. Fig. 1

Suppl. Fig. 2

Supplement: Supplementary file 1 — Supplementary Material 1. [file 13005_2026_618_MOESM1_ESM.docx]
